# A systematic comparison of the MetaCyc and KEGG pathway databases

**DOI:** 10.1186/1471-2105-14-112

**Published:** 2013-03-27

**Authors:** Tomer Altman, Michael Travers, Anamika Kothari, Ron Caspi, Peter D Karp

**Affiliations:** 1 Bioinformatics Research Group, SRI International, Menlo Park, USA

**Keywords:** Pathway databases, Database comparison

## Abstract

**Background:**

The MetaCyc and KEGG projects have developed large metabolic pathway databases that are used for a variety of applications including genome analysis and metabolic engineering. We present a comparison of the compound, reaction, and pathway content of MetaCyc version 16.0 and a KEGG version downloaded on Feb-27-2012 to increase understanding of their relative sizes, their degree of overlap, and their scope. To assess their overlap, we must know the correspondences between compounds, reactions, and pathways in MetaCyc, and those in KEGG. We devoted significant effort to computational and manual matching of these entities, and we evaluated the accuracy of the correspondences.

**Results:**

KEGG contains 179 module pathways versus 1,846 base pathways in MetaCyc; KEGG contains 237 map pathways versus 296 super pathways in MetaCyc. KEGG pathways contain 3.3 times as many reactions on average as do MetaCyc pathways, and the databases employ different conceptualizations of metabolic pathways. KEGG contains 8,692 reactions versus 10,262 for MetaCyc. 6,174 KEGG reactions are components of KEGG pathways versus 6,348 for MetaCyc. KEGG contains 16,586 compounds versus 11,991 for MetaCyc. 6,912 KEGG compounds act as substrates in KEGG reactions versus 8,891 for MetaCyc. MetaCyc contains a broader set of database attributes than does KEGG, such as relationships from a compound to enzymes that it regulates, identification of spontaneous reactions, and the expected taxonomic range of metabolic pathways. MetaCyc contains many pathways not found in KEGG, from plants, fungi, metazoa, and actinobacteria; KEGG contains pathways not found in MetaCyc, for xenobiotic degradation, glycan metabolism, and metabolism of terpenoids and polyketides. MetaCyc contains fewer unbalanced reactions, which facilitates metabolic modeling such as using flux-balance analysis. MetaCyc includes generic reactions that may be instantiated computationally.

**Conclusions:**

KEGG contains significantly more compounds than does MetaCyc, whereas MetaCyc contains significantly more reactions and pathways than does KEGG, in particular KEGG modules are quite incomplete. The number of reactions occurring in pathways in the two DBs are quite similar.

## Background

MetaCyc [1-8] and KEGG [9-15] are large metabolic pathway database (DB) projects that have been under development for more than a decade. Both projects provide reference pathways that are used to predict the metabolic pathways present in an organism from the annotated genome of that organism. MetaCyc has been utilized for pathway prediction in the BioCyc database collection [2], and in many other Pathway/Genome Databases developed by researchers around the world [16]. The KEGG project consists of both a reference pathway database, and the resulting projection of the reference pathways onto organisms with sequenced genomes. KEGG is also widely used.

The goal of this article is to compare the data content of MetaCyc with the data content of the KEGG reference pathway DB to provide an understanding of their relative sizes, their degree of overlap, their scope, and the breadth of data that they provide. These questions are particularly important because the accuracy of metabolic pathway prediction is directly dependent upon the coverage of the reference pathway DB that the pathway predictor utilizes [17]. Most metabolic pathway predictors predict the presence of pathways that exist in a reference pathway DB. Another important and active area of research is the development of steady-state metabolic flux models. These models are highly dependent upon the source of metabolic reactions from which the models are derived. Our analysis considers KEGG and MetaCyc because they are the largest curated databases of metabolic reactions and pathways (see Table 2 in [18]), containing significantly more reactions than Rhea, BiGG, UniPathway, BioPath, and Reactome. Although The SEED and BRENDA contain comparable numbers of reactions, the metabolic content of The SEED is largely derived from KEGG [19], and BRENDA does not include metabolic pathways.

Metabolic pathway data can be thought of as consisting of three tiers: the metabolites form the lowest tier; reactions are built upon metabolites, and pathways are built upon reactions. Our analysis considers all three tiers. Our comparison does not include other aspects of KEGG or BioCyc, such as their orthology data, or their genome-based pathway predictions for sequenced organisms.

To analyze the degree of overlap of MetaCyc and KEGG, we must know the correspondences between compounds, reactions, and pathways in MetaCyc, and those in KEGG. Establishing these correspondences is a non-trivial problem because of the non-standard terminologies used in the scientific literature for these three entities (e.g., large numbers of synonyms are used for a given chemical compound), because some metabolites lack chemical structures in one DB or the other, and because stereochemical information is not present for every metabolite that contains a stereo center, thus precluding complete matching using chemical structures. We devoted significant effort to the problem of matching metabolites and reactions (see Methods). As a result, many MetaCyc compounds and reactions now contain DB links to the corresponding objects in KEGG. MetaCyc pathways do not contain such links because metabolic pathways have more subjective definitions than do metabolites and reactions, and hence we do not expect there to be exact correspondences between the pathways in MetaCyc and KEGG.

Consider the following problem that is common to this study, to other recent studies that compare bioinformatics DBs [20,21], and to efforts that integrate data from multiple metabolic DBs [18,22-25]. To compare, for example, the metabolite complements of two pathway DBs, we must know which metabolites within the DBs correspond to one another. But the algorithms that compute such correspondences are imperfect. If metabolite *M* in one DB is found to have no counterpart in the other DB, does this observation reflect the true state of affairs, or a false-negative result by the matching algorithm? We present a method for approaching this problem — namely, to sample matched and unmatched objects from the DBs and manually validate or search for correspondences to quantify the accuracy of the correspondences. A *caveat* for all of our analyses is that they are dependent on the accuracy and completeness of the compound and reaction correspondences that we have curated, imported, and computed. All curated, imported, and inferred links from MetaCyc compounds and reactions to their corresponding entries in KEGG may be found in the MetaCyc files that are available for download from the web.

We also introduce a general method for assessing where is the semantic overlap between two DBs: we use enrichment/depletion analysis to detect whether areas of one DB are disproportionately populated or depopulated with respect to the other DB.

## Methods

We used MetaCyc version 16.0 (released on February 17, 2012) and a version of KEGG downloaded on February 27, 2012 for the purpose of this study, unless otherwise noted. The KEGG datasets were downloaded using the KEGG SOAP object-retrieval web services [26] using the following functions *via* the BioBike software [27]: list_organisms, binfo, list_pathways, bfind, bget, get_compounds_by_pathway, get_ reactions_by_pathway, and get_element_rela‐ tions_by_pathway.

The KEGG data were loaded into a new Pathway Tools [28] DB (“KeggCyc”) that uses the same schema as MetaCyc. The loaded KEGG datasets were COMPOUND, REACTION, MAP, and MODULE. KEGG chemical structures were obtained from the COMPOUND dataset, not from KEGG MOL files. This KEGG loader was implemented in Common Lisp, and is available in the Additional file 1. Once loaded into a Pathway Tools DB, the KEGG data can be queried and visualized using Pathway Tools. The KEGG analyses reported herein were performed using Common Lisp programs that queried KeggCyc.

### Comparing compound data

Correspondences between MetaCyc compounds and KEGG compounds are encoded as MetaCyc compounds DB links to the corresponding KEGG compound. Such DB links are added to MetaCyc by several means. The MetaCyc curation staff members add such links during their manual curation. In addition, we submit MetaCyc compounds with chemical structures to the PubChem standardization pipeline in order to match MetaCyc compound structures with PubChem Compound entries. KEGG compounds are also periodically processed by the same PubChem standardization pipeline. We have imported links to KEGG compounds from PubChem Compound dataset that are linked to both MetaCyc and KEGG compounds. We also received compound correspondences between MetaCyc and KEGG compounds from other research efforts, which we checked for errors before programmatically importing them into MetaCyc (John Bates, Dylan Chivan, personal communication).

We implemented a rule-based system to predict additional compound matches between MetaCyc and KEGG. We defined a set of compound match features, such that a proposed compound match required one or more features from the set, along with a compound name exact string match, in order for a match to be predicted. We defined the set of compound features for MetaCyc and KEGG compounds as molecular fingerprint matching *via* PubChem [29] coupled with the Pathway Tools compound structure matcher, exact stereo-structure matching, and “all-but-one” inference. We utilized the pre-computed molecular fingerprints for KEGG and MetaCyc compounds in PubChem to detect compound pairs that had a Tanimoto coefficient [30] greater than 0.75. We then further filtered the compound pairs by using the compound structure matcher from Pathway Tools, which can detect compounds with the same structure, even if there are differences in protonation state.

“All-but-one” compound match inference is where a pair of known corresponding reactions, one from MetaCyc and the other from KEGG, have all of their substrates matched except for one pair of substrates — *C*_*M*_ from the MetaCyc reaction and *C*_*K*_ from the KEGG reaction. If *C*_*M*_ does not already have a compound link to a KEGG compound, and *C*_*K*_ does not already have a compound link to a MetaCyc compound, we infer a match between *C*_*M*_ and *C*_*K*_.

For the exact string matching of the compound common name, name strings were “canonicalized” to remove differences such as punctuation and capitalization prior to checking for exact matches. Any pair of compounds consisting of a MetaCyc compound and a KEGG compound that had at least one feature match from the set, had an exact string match of their names, and had no contradictory matches (e.g., where our rule-based system inferred that a single MetaCyc compound had two matching KEGG compounds) were inferred as compound matches. Automatically inferred compound matches were randomly sampled for review by our curation staff for quality assurance. 1214 compound correspondences were inferred using this system.

The International Union of Pure and Applied Chemistry International Chemical Identifier (InChI) is a method of generating a unique string representation of a chemical compound structure [31]. We used the official InChI software package (version 1.02) for generating standard InChI strings from compounds with structures in MetaCyc and KEGG. The InChI executable was called with the following arguments: ‐STDIO ‐NoLabels ‐AuxNone. We infer two compounds as having matching structure if their standard InChI strings are identical. We used InChI strings to detect compound matches between MetaCyc and KEGG, and to detect duplicate compounds within MetaCyc or KEGG. InChI strings may be used to detect differences in stereo-center orientation between two compounds that otherwise have the same structure. Both KEGG and MetaCyc contain compounds with one or more unspecified stereo centers, and thus using InChI strings to detect matching compounds may miss some legitimate matches. Furthermore, InChI strings are different for two different protonation states [1] of the same compound, and thus equivalent compounds with different protonation states between KEGG and MetaCyc may be overlooked by comparing InChI strings. For related work on establishing correspondences between metabolic databases using string matching, see MetRxn [23] (which matches metabolites on a canonical SMILES structure representation) and BKM-react [24] (which matches metabolites on InChI string and name).

We assessed the accuracy of our correspondences between KEGG and MetaCyc compounds. Specifically, we applied the binomial distribution to estimate the proportion of false negatives at a confidence level of 90% and with a confidence interval of 10%, which indicated that 68 samples were necessary. Thus, we sampled 68 MetaCyc compounds with no inferred link to a compound in KEGG and manually searched for a corresponding KEGG compound. We found 9 MetaCyc compounds (13.2%) that did have a corresponding entry in KEGG. This result implies that we have 90% confidence that the true proportion of false negative predictions is between 0.0013 and 0.232, meaning that as many as 1650 MetaCyc compounds remain to have their corresponding KEGG compound determined. We used a separate sampling of 68 MetaCyc compounds with inferred links to KEGG compounds in order to estimate the proportion of false positives at the same confidence level and interval. Manual verification of the correspondences identified one compound (1.5%) that had an incorrect correspondence. This implies that we have 90% confidence that the true proportion of false positive predictions is between 8.4×10^−4^ and 11.5%, or as many as 140 compounds with incorrect correspondences. With the false positive and false negative rates estimated, the overall accuracy is 85.3%, with an upper bound of 99.9% and a lower bound of 65.3% for the given confidence level and interval.

In addition to reporting the number of compounds in the intersection between MetaCyc and KEGG for various compound categories, we compute the Jaccard coefficient as a measure of the similarity between the two sets. The Jaccard coefficient can be simply defined as the size of the intersection of the two sets divided by the size of the union of the two sets [30]. Thus, two sets that are identical will have a Jaccard coefficient of one, while two disjoint sets will have a Jaccard coefficient of zero. We use the Jaccard coefficient in comparing the overlap of reactions in MetaCyc and KEGG as well.

### Comparing reaction data

The KEGG COMPOUND dataset contains entries that are pharmaceuticals or glycan compounds, and thus have matching entries in the KEGG DRUG and GLYCAN datasets, respectively. These objects are designated with “D” and “G” prefixes in their identifiers and are duplicates of the standard compound objects, in the COMPOUND dataset, that use an identifier prefix of “C”. We found that the KEGG REACTION dataset contains duplicate reactions, where one version of a reaction will use the “C” identifiers, while the other version of the same reaction would use the “G” or “D” identifiers. We analyzed only reactions that consisted of “C” identifiers for the purpose of this study to avoid double-counting reactions.

We used a combination of manual curation, a computational rule-based system for inferring reaction correspondences, and bulk extraction of reaction correspondences from databases such as BKM-React, GO, MetRxn, and Rhea [23,24,32,33] to create links between MetaCyc and KEGG reactions.

A proposed reaction match required a significant similarity of reactants and products, along with at least one match from a set of three reaction features. The first feature from the set checked the enzymes that catalyzed the pair of reactions from MetaCyc and KEGG to see if they had at least one UniProt [34] accession number in common. The second feature employed exact matches of International Union of Biochemistry and Molecular Biology (IUBMB) Enzyme Commission (EC) [35] enzyme classification number matches. The third feature checked for exact enzymatic activity name matches, using KEGG REACTION entry names and enzymatic activity names from MetaCyc enzyme information associated with reaction objects. Reaction and enzymatic activity name strings were “canonicalized” to remove differences such as punctuation and capitalization prior to checking for exact matches.

To detect similarities of reaction reactants and products, we defined a feature by representing reactions as column vectors in a stoichiometric matrix [36]. In brief, a stoichiometric matrix represents reactions as columns, and each distinct compound in the metabolic network is represented by a row. Matrix values are zero unless a particular compound participates in a particular reaction, in which case the coefficient of the compound in the reaction is inserted at the corresponding row and column in the matrix. Reactant coefficients are entered into the matrix as negative values; product coefficients are positive.

We inferred a match if the absolute value of the cosine similarity [30] of the MetaCyc stoichiometry vector and the KEGG stoichiometry vector was greater than 0.6 (with identical reactions having a cosine similarity of 1.0), along with the reaction pair having one or more matches from the set of three reaction match features, and no contradictory matches. Finally, any remaining pairs of reactions between KEGG and MetaCyc that had no other matches, yet had the same EC number, and the EC number based matching was one-to-one, were inferred as matches. 3211 reaction object correspondences were inferred using this system.

We sought to assess the accuracy of our reaction correspondences using a sampling procedure. As described for compounds, we sampled 68 MetaCyc reactions with no inferred link to a reaction in KEGG and 68 MetaCyc reactions with an inferred link to a reaction in KEGG. We found 9 MetaCyc reactions without links (13.2%) that did have a corresponding entry in KEGG. This implies that we have 90% confidence that the true proportion of false negative predictions is between 0.0013 and 0.232, or as many as 1636 MetaCyc reactions remaining to have their corresponding KEGG reaction determined. We found 6 reactions (8.8%) that had an incorrect link. This implies that we have 90% confidence that the true proportion of false positive predictions is between.0018 and 0.188, or as many as 603 reactions with incorrect links. With the false positive and false negative rates estimated, the overall accuracy is 78.0%, with an upper bound of 99.69% and a lower bound of 58% for the given confidence level and interval.

To assess the balance state of reactions in MetaCyc and KEGG we used the reaction balance checker in Pathway Tools. This software counts the atoms present on both sides of a given reaction and checks for equality of atom counts. Although MetaCyc compounds are protonated consistently relative to a defined pH [37], we are not aware of any consistent protonation among KEGG compounds. To avoid unduly penalizing KEGG reactions, we also checked the balance state of reactions in both MetaCyc and KEGG for all atoms aside from hydrogen.

### Comparing pathway data

MetaCyc contains two types of pathways: *base pathways* are individual metabolic pathways (example: TRPSYN‐PWY, “tryptophan biosynthesis”); *super pathways* combine sets of base pathways, super pathways, and individual reactions into larger composite pathways (example: ALL‐CHORISMATE‐PWY, “superpathway of chorismate metabolism”). Similarly, KEGG contains analogous notions of *modules* and *maps*. KEGG modules were introduced to “define tighter functional units than KEGG PATHWAY” [38]. The KEGG pathway data were obtained from the MODULE and MAP datasets. KEGG modules are of four types: pathway modules, structural protein and RNA complexes, functional sets, and signature modules. The latter three types of modules are not metabolic pathways and are excluded from our analyses, thus excluding more than half of KEGG modules. KEGG defines three “Global Pathways” in the MAP dataset: map01100 (“Metabolic Pathways”), map01120 (“Microbial metabolism in diverse environments”), and map01110 (“Biosynthesis of secondary metabolites”). These pathways include thousands of reactions from many other maps. Since these three large maps are qualitatively different entities than the other KEGG maps, we excluded them from the analysis herein. Furthermore, KEGG pathway classes where none of the associated pathway instances have any metabolic reaction data (such as the “Environmental information processing” pathway class) were also excluded since they do not contain metabolic pathway data.

## Results

### Compound comparison

Table 1 compares the number of chemical compounds found in MetaCyc and KEGG. Table 2 compares the types of compound data present in KEGG and MetaCyc, and the degree to which different data fields are populated in the two DBs.

**Table 1:** Comparison of chemical compounds in MetaCyc and KEGG

**Category**	**M(all)**	**M(base)**	**M(super)**	**K(all)**	**K(module)**	**K(map)**	**Common**
All chemical compounds	11991			15161			5120 (0.23)
All reaction substrates	8891			6912			4232 (0.37)
Pathway reaction substrates	5523	5371	5523	4759	828	4759	2384 (0.30)

For each type of compound (row), we report the number of compounds in MetaCyc, the number of compounds in KEGG, and the number of compounds in common between MetaCyc and KEGG. “All chemical compounds” includes both compound classes and compound instances for MetaCyc; for KEGG it includes all compounds in the KEGG COMPOUND file. “All reaction substrates” is the union of all literal reaction substrates (reactants plus products) in the specified DB. M(all): all MetaCyc compounds; M(base): compounds in MetaCyc base pathways; M(super): compounds in MetaCyc super pathways; K(all): all KEGG compounds; K(module): compounds in KEGG module; K(map): compounds in KEGG map; Common: corresponding compounds by total number and by the Jaccard coefficient in parentheses.

**Table 2:** Comparison of compound data content in MetaCyc and KEGG

	**MetaCyc**	**KEGG**
Compounds	11991	15161
Compounds with structures	10546	14621
Compounds with comments	1486	2997
Mean comment length	47.69	6.51
Mean names per compound	2.37	1.62
Mean DB links per compound	1.71	3.71
Mean associated reactions	3.59	2.17
Mean associated pathways (all) per compound	1.78	0.67
Duplicate compounds	36	251

Compound entries in either DB may not have information on their chemical structures, and may not have comments describing the properties of the compound. Associated pathways of a compound include base pathways and superpathways in MetaCyc and KEGG maps and modules. Compounds were considered duplicates if they had identical standard InChI strings.

Table 3 compares additional MetaCyc and KEGG compound object attributes. The table omits KEGG compound attributes called ENZYME and PATHWAY, which represent relationships between KEGG objects. The equivalent relationships can easily be extracted from MetaCyc *via* other objects and attributes, and thus are not represented as direct attributes of the compounds.

**Table 3:** A comparison of MetaCyc and KEGG compound attributes, for those attributes where one hundred or more objects have a value for that attribute

**MetaCyc**		**KEGG**	
**Attribute**	**Frequency**	**Attribute**	**Frequency**
Monoisotopic-MW	9475	Exact_Mass	14611
Molecular-Weight	9431	Mol_Weight	14611
Creation-date	11705		
Creator	10573		
SMILES	10546		
InChI	9222		
Regulates	3573		
Credits	2895		
Gibbs-0	1033		
Cofactors-Of	563		

The table presents shared attributes (note that the name for the same conceptual attribute may differ between MetaCyc and KEGG), and attributes unique to MetaCyc; the two attribute sets are further sorted based on the number of objects containing non-null values for each attribute. Gibbs-0 is the Gibbs free energy of formation of a compound. Creator, Creation-Date, and Credits provide data provenance. KEGG compound attribute data are derived from the KEGG COMPOUND dataset.

### Reaction comparison

Table 4 compares the number of reactions between MetaCyc and KEGG. Tables 5 and 6 compare different aspects of reaction attributes between MetaCyc and KEGG. Note that KEGG provides its data in a number of other formats. For example, KEGG KGML files contain information on reaction reversibility, but because our study is limited to data accessible *via* the SOAP web services, that reversibility information is not listed in Table 6.

**Table 4:** Comparison of biochemical reactions in MetaCyc and KEGG

**Category**	**M(all)**	**M(base)**	**M(super)**	**K(all)**	**K(module)**	**K(map)**	**Common**
All reactions	10262			8692			3895 (0.26)
Pathway reactions	6348	6155	6348	6174	878	6173	1961 (0.19)

Mens are pathway reactions if they are part of one or more base pathways or superpathways. KEGG reactions are pathway reactions if they are part of one or more modules or maps. Columns are the same as defined for Table 1.

**Table 5:** Comparison of reaction data content in MetaCyc and KEGG

**Category**	**MetaCyc**	**KEGG**
Reaction instances	10262	8879
Duplicate reactions	279	341
Reactions with comments	3206	3022
Unbalanced reactions (not counting hydrogen)	474	872
Unbalanced reactions (counting hydrogen)	532	1475
Mean associated pathways	0.84	0.90

We report the number of reactions, the number of duplicate reactions, the number of reactions with comments, the number of unbalanced reactions disregarding hydrogen imbalance, the number of unbalanced reactions including hydrogen imbalance, and the average number of associated pathways. Associated pathways of a reaction include base pathways and superpathways in MetaCyc and KEGG modules and maps.

**Table 6:** A comparison of MetaCyc (M) and KEGG (K) reaction attributes, for those attributes where one hundred or more objects have a value for that attribute

**MetaCyc**		**KEGG**	
**Attribute (M)**	**Frequency (M)**	**Attribute (K)**	**Frequency (K)**
Physiologically-Relevant?	10262		
Creation-Date	10247		
		Rpair	8292
Creator	8090		
EC-Number	7998	Enzyme	7632
Reaction-Direction	6660		
Orphan?	5967		
Credits	2779		
Rxn-Locations	282		
Spontaneous?	238		

Attributes are sorted based on the MetaCyc frequency column. Attribute Physiologically-Relevant? describes whether a reaction occurs *in vivo*. Reaction-Direction specifies the directionality of the reaction. Orphan? is true when no nucleotide or amino-acid sequence has been determined for any enzyme catalyzing this reaction [39,40]. Rxn-Locations specifies the cellular locations in which a reaction occurs (e.g., cytoplasm or mitochondrion). Spontaneous? specifies whether a reaction occurs spontaneously in living organisms and therefore requires no enzyme. KEGG reaction attribute data are derived from the KEGG REACTION dataset, and thus include glycan reactions.

Since hydrogen imbalance in KEGG reactions can be the result of inconsistent protonation states of the compounds involved, we consider such imbalance as a potentially less serious problem than imbalance by other elements. Thus, we conducted a special set of analyses for reaction balancing where we did not count hydrogen atoms.

Two reactions *R*_1_ and *R*_2_ are duplicates if the reactants of *R*_1_ are the same as the reactants of *R*_2_, and the products of *R*_1_ are the same as the products of *R*_2_, or ditto for the reverse of *R*_1_.

### Pathway comparison

A summary of MetaCyc base and super pathways, and KEGG modules and maps is presented in Table 7. A comparison of pathway data in MetaCyc and KEGG is presented in Table 8.

**Table 7:** Comparison of metabolic pathways, average reactions per pathway, and average compounds per pathway in MetaCyc (M) and KEGG (K)

**Category**	**M(base)**	**K(module)**	**M(super)**	**K(map)**
Pathway count	1846	179	296	237
Reactions perpathway	4.37	6.22	14.24	28.84
Compounds per pathway	11.49	15.27	25.63	37.45

**Table 8:** Comparison of pathway data content in MetaCyc and KEGG

**Category**	**MetaCyc**	**KEGG**
Pathway classes	490	107
Pathway instances	2142	416
Pathways with comments	2122	51
Mean comment length	2240.6	83.6
DB links per pathway	0.34	0.88
Reactions per pathway	5.73	19.10

KEGG pathway classes were extracted from the MAP and MODULE datasets based on the CLASS attribute. Comment length is measured in number of characters.

**Figure 1:**
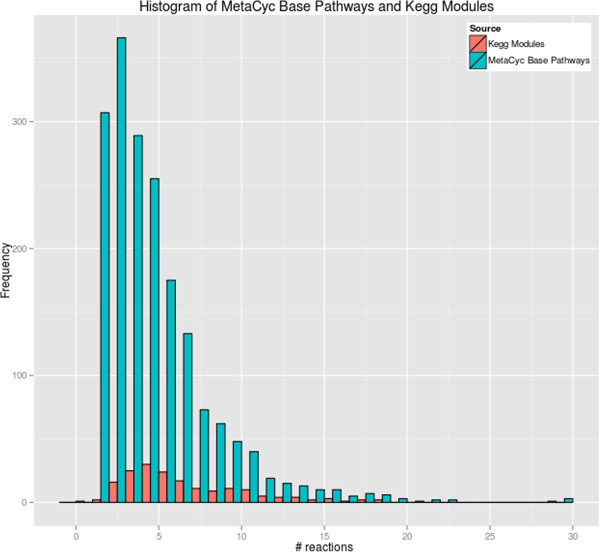
**A histogram plot of MetaCyc base pathway and KEGG module size by reaction counts.** We excluded one outlier consisting of a MetaCyc base pathway (PWYG-321, “mycolate biosynthesis”) with 192 reactions; 17% of MetaCyc base pathways consist of a single reaction.

**Figure 2:**
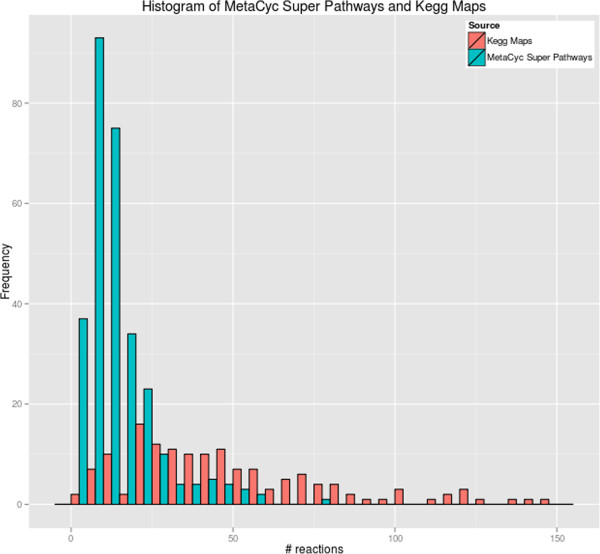
**A histogram plot of MetaCyc super pathway and KEGG map size by reaction counts.** We excluded one outlier consisting of a MetaCyc super pathway (PWY-6113, “mycolate biosynthesis”) with 233 reactions.

A histogram plot of the frequency of MetaCyc base pathway sizes (by reaction count) and KEGG modules sizes (by reaction count) is presented in Figure 1, and a histogram plot of the frequency of MetaCyc super pathways and KEGG maps sizes by reaction count is presented in Figure 2.

See Table 9 for a comparison of other MetaCyc and KEGG pathway object attributes. Note that the KEGG module attribute called COMPOUND links compound objects with modules. In MetaCyc the equivalent relationship is represented *via* reaction objects, and thus there is no need for an additional attribute.

**Table 9:** A comparison of MetaCyc (M) and KEGG (K) pathway attributes, for those attributes where one hundred or more objects have a value for that attribute

**MetaCyc**		**KEGG**	
**Attribute (M)**	**Frequency (M)**	**Attribute (K)**	**Frequency (K)**
Species	2141		
Pathway-Links	1412	Rel_Pathway	345
Creation-Date	2139		
Taxonomic-Range	2135		
Creator	2092		
Predecessors	2089	ECrel	154
Credits	1944		
Key-Reactions	373		
		Disease	220
Hypothetical-Reactions	105		

Attributes are sorted based on frequency. KEGG pathway attribute data are pooled from all objects in the KEGG MODULE and MAP datasets (which include data from global pathways and pathway classes with no metabolic reaction data). Attribute Species specifies the organisms in which the pathway has been studied experimentally. Pathway-Links lists important substrates that connect to other metabolic pathways, whereas KEGG attribute Rel_Pathway links pathways to one another without specifying the compound in common. Taxonomic-Range describes the taxonomic groups in which the pathway is likely to be found; this information increases the accuracy of pathway prediction. Predecessors specifies for each reaction the reaction(s) that precede it in the pathway, and thus defines the connectivity structure of each pathway. KEGG encodes equivalent data in the “ECrel” relationship, obtained from the get_element_relations_by_pathway API function. Key-Reactions increases the accuracy of pathway prediction by specifying reactions whose presence is highly indicative of the pathway, and distinguish the pathway from other, similar pathways. Hypothetical-Reactions identifies pathway reactions that are speculative and have not been firmly established experimentally. The Disease attribute consists of links to the KEGG DISEASE dataset when disease-related genes encode enzymes for one or more reaction steps in the pathway.

We next sought to understand how much KEGG and MetaCyc pathways overlap, a question that we approach from several directions. Table 10 explores the degree to which KEGG and MetaCyc pathways cover their respective reaction spaces. For example, if a KEGG module *M* contains six reactions, and none of those reactions are present in (linked to) MetaCyc (based on the MetaCyc reaction links to KEGG reactions), we think of *M* as unique to KEGG because it covers a part of reaction space that is not present in MetaCyc. We say that a pathway class *C* is enriched/depleted for reaction links if the reactions *R*_*i*_ contained within pathway instances *P*_*j*_ in *C*, are enriched/depleted (as described in Section ‘Pathway comparison’) for links to reactions in KEGG. Such analysis is, of course, limited by the fact that our reaction links are imperfect. Table 10 summarizes the fraction of MetaCyc pathways for which all, some, or none of their reactions correspond to reactions in KEGG, and the converse — the fraction of KEGG pathways for which all, some, or none of their reactions correspond to reactions in MetaCyc. MetaCyc pathways include a total of 3,901 reactions not found in KEGG, whereas KEGG pathways include a total of 3,852 reactions not found in MetaCyc.

**Table 10:** Degree to which pathways in MetaCyc (M) and KEGG (K) have their reactions linked to the other DB

**ReactionCoverage**	**M(base)**	**K(module)**	**M(super)**	**K(map)**
All reactionslinked	549	73	0	3
Some reactionslinked	731	80	73	128
No reactionslinked	566	26	223	106

For example, for three KEGG maps, all reactions in the pathway are present in MetaCyc.

Another way to address the degree of overlap of their pathways is through the MetaCyc pathway ontology. We performed enrichment/depletion analysis in order to determine pathway classes that had sets of matched reactions that were significantly smaller or larger than what might be expected by chance [41]. For determining the pathway classes of MetaCyc that were significantly enriched or depleted for reactions linked to KEGG reactions, we used the Pathway Tools Enrichment Analysis feature. Specifically, we ran the Enrichment Analysis using the exact Fisher method with a maximum p-value of 0.025 and employing the Bonferroni multiple hypothesis testing correction. The results are summarized in Table 11 for MetaCyc pathway classes, and in Table 12 for KEGG pathway classes. Complete results are available in Additional file 2.

**Table 11:** MetaCyc pathway classes that are significantly enriched or depleted for reactions with links to KEGG

**Status**	**Pathway class**	**Class size**	**Links**	**p-value**
Enriched	Amino Acids Biosynthesis	112	$\frac{186}{260}$	1.4 × 10^−20^
Enriched	Individual Amino Acids Biosynthesis	99	$\frac{174}{244}$	4.0 × 10^−19^
Enriched	Amino Acids Degradation	118	$\frac{222}{326}$	2.0 × 10^−17^
Enriched	Purine Nucleotide Biosynthesis	19	$\frac{46}{56}$	3.2 × 10^−10^
Enriched	Generation Of Precursor Metabolites And Energy	162	$\frac{170}{304}$	2.6 × 10^−9^
Enriched	C1 Compounds Utilization And Assimilation	28	$\frac{75}{102}$	9.3 × 10^−9^
Enriched	Autotrophic CO_2_ Fixation	7	$\frac{47}{57}$	1.3 × 10^−7^
Enriched	CO_2_ Fixation	9	$\frac{48}{59}$	2.8 × 10^−7^
Enriched	Vitamins Biosynthesis	68	$\frac{128}{223}$	1.4 × 10^−6^
Enriched	Sugar Derivatives Degradation	42	$\frac{109}{162}$	3.8 × 10^−6^
Enriched	Sugar Alcohols Degradation	12	$\frac{54}{68}$	6.0 × 10^−6^
Enriched	Amines And Polyamines Biosynthesis	37	$\frac{57}{74}$	6.9 × 10^−6^
Enriched	Carboxylates Degradation	44	$\frac{82}{132}$	1.0 × 10^−5^
Enriched	Sugars Degradation	51	$\frac{108}{162}$	1.5 × 10^−5^
Enriched	NAD Biosynthesis	8	$\frac{23}{23}$	1.6 × 10^−5^
Enriched	Fermentation	46	$\frac{75}{106}$	3.6 × 10^−5^
Enriched	Nucleosides And Nucleotides Biosynthesis	35	$\frac{69}{128}$	6.8 × 10^−5^
Enriched	Nucleosides And Nucleotides Degradation	29	$\frac{64}{90}$	1.4 × 10^−4^
Enriched	Purine Nucleotide Salvage	13	$\frac{26}{28}$	1.7 × 10^−4^
Enriched	Arginine Degradation	15	$\frac{35}{42}$	4.6 × 10^−4^
Enriched	Purine Nucleotide De Novo Biosynthesis	6	$\frac{22}{30}$	4.9 × 10^−4^
Enriched	Mandelates Degradation	2	$\frac{18}{18}$	9.9 × 10^−4^
Enriched	Gluconeogenesis	2	$\frac{23}{28}$	1.1 × 10^−3^
Enriched	Glycolysis	6	$\frac{27}{30}$	2.0 × 10^−3^
Enriched	NAD Metabolism	11	$\frac{28}{33}$	2.1 × 10^−3^
Enriched	Geranylgeranyldiphosphate Biosynthesis	3	$\frac{18}{18}$	2.3 × 10^−3^
Enriched	Catechol Degradation	7	$\frac{17}{17}$	2.3 × 10^−3^
Enriched	Methionine Biosynthesis	13	$\frac{29}{34}$	4.0 × 10^−3^
Enriched	Photosynthesis	5	$\frac{24}{30}$	4.0 × 10^−3^
Enriched	Pyrimidine Nucleotide Biosynthesis	8	$\frac{36}{53}$	5.7 × 10^−3^
Enriched	Toluenes Degradation	13	$\frac{35}{46}$	7.2 × 10^−3^
Enriched	Glutamate Degradation	10	$\frac{28}{35}$	1.5 × 10^−2^
Enriched	Formaldehyde Assimilation	3	$\frac{24}{28}$	1.6 × 10^−2^
Enriched	Alcohols Degradation	17	$\frac{24}{30}$	1.6 × 10^−2^
Enriched	Urate Degradation	2	$\frac{17}{18}$	2.4 × 10^−2^
Enriched	Cobalamin Biosynthesis	9	$\frac{35}{46}$	2.5 × 10^−2^
Depleted	Secondary Metabolites Biosynthesis	460	$\frac{579}{1896}$	3.8 × 10^−35^
Depleted	Glucosinolates Biosynthesis	9	$\frac{4}{104}$	2.0 × 10^−17^
Depleted	Biosynthesis	1182	$\frac{1459}{4215}$	2.3 × 10^−16^
Depleted	Nitrogen Containing Glucosides Biosynthesis	13	$\frac{11}{125}$	8.0 × 10^−15^
Depleted	Hormones Degradation	24	$\frac{12}{124}$	2.7 × 10^−13^
Depleted	Polymeric Compounds Degradation	35	$\frac{17}{136}$	3.5 × 10^−12^
Depleted	Polysaccharides Degradation	33	$\frac{17}{127}$	2.0 × 10^−10^
Depleted	Steroids Degradation	8	$\frac{2}{46}$	4.2 × 10^−6^
Depleted	Polyketides Biosynthesis	13	$\frac{6}{62}$	1.5 × 10^−5^
Depleted	Glucosinolates Degradation	4	$\frac{0}{30}$	7.0 × 10^−5^
Depleted	Cholesterol Degradation	4	$\frac{1}{33}$	3.3 × 10^−4^
Depleted	Fatty Acid Biosynthesis	49	$\frac{20}{354}$	3.4 × 10^−4^
Depleted	Nitrogen Containing Secondary Compounds Degradation	18	$\frac{13}{77}$	1.0 × 10^−3^
Depleted	Terpenoids Biosynthesis	127	$\frac{176}{530}$	1.2 × 10^−3^
Depleted	Plant Hormones Degradation	15	$\frac{7}{55}$	1.6 × 10^−3^
Depleted	Sesquiterpenoids Biosynthesis	32	$\frac{25}{114}$	1.6 × 10^−3^
Depleted	Chlorotoluene Degradation	5	$\frac{0}{24}$	2.3 × 10^−3^
Depleted	Auxins Degradation	8	$\frac{0}{23}$	4.1 × 10^−3^
Depleted	Apocarotenoids Biosynthesis	4	$\frac{0}{20}$	2.4 × 10^−2^
Depleted	Lignans Biosynthesis	5	$\frac{0}{20}$	2.4 × 10^−2^

Class size is the number of pathway instances for the given pathway class. The ‘Links’ column is the number of reactions among the pathways of the pathway class that have links to KEGG reactions, over the total number of reactions for the pathway class. The Bonferroni-corrected p-value from the hypergeometric test indicates the probability that the observed proportion of reactions with links within the pathway occurred by chance. Pathways with a p-value at or below a cut-off of *α* = 0.025 are shown. The full list may be found in the Additional file 2.

**Table 12:** KEGG pathway classes that are significantly enriched or depleted for reactions with links to MetaCyc

**Status**	**Pathway class**	**Class size**	**Links**	**p-value**
Enriched	Nucleotide And Amino Acid Metabolism	72	$\frac{346}{419}$	2.6 × 10^−56^
Enriched	Carbohydrate Metabolism	15	$\frac{496}{766}$	4.1 × 10^−29^
Enriched	Amino Acid Metabolism	13	$\frac{478}{784}$	3.0 × 10^−20^
Enriched	Energy Metabolism	8	$\frac{167}{246}$	2.9 × 10^−14^
Enriched	Cofactor And Vitamin Biosynthesis	19	$\frac{104}{133}$	1.8 × 10^−11^
Enriched	Energy Metabolism	24	$\frac{89}{114}$	1.3 × 10^−10^
Enriched	Carbon Fixation	13	$\frac{56}{66}$	7.6 × 10^−8^
Enriched	Aromatic Amino Acid Metabolism	11	$\frac{45}{55}$	2.2 × 10^−5^
Enriched	Alkaloid And Other Secondary Metabolite Biosynthesis	4	$\frac{28}{30}$	2.9 × 10^−5^
Enriched	Other Carbohydrate Metabolism	6	$\frac{37}{44}$	2.9 × 10^−5^
Enriched	Nucleotide Metabolism	2	$\frac{159}{261}$	7.6 × 10^−5^
Enriched	Cysteine And Methionine Metabolism	6	$\frac{27}{30}$	3.4 × 10^−4^
Enriched	Central Carbohydrate Metabolism	13	$\frac{42}{52}$	4.4 × 10^−4^
Enriched	Reaction Motif	3	$\frac{15}{15}$	7.1 × 10^−3^
Enriched	Arginine And Proline Metabolism	3	$\frac{15}{15}$	7.1 × 10^−3^
Enriched	Histidine Metabolism	2	$\frac{14}{14}$	1.6 × 10^−2^
Enriched	Purine Metabolism	3	$\frac{23}{28}$	2.1 × 10^−2^
Depleted	Xenobiotics Biodegradation And Metabolism	20	$\frac{258}{1013}$	1.0 × 10^−37^
Depleted	Glycan Biosynthesis And Metabolism	15	$\frac{36}{254}$	1.9 × 10^−21^
Depleted	Metabolism Of Terpenoids And Polyketides	20	$\frac{265}{848}$	4.4 × 10^−13^
Depleted	Glycan Metabolism	10	$\frac{0}{51}$	6.8 × 10^−11^
Depleted	Glycosaminoglycan Metabolism	7	$\frac{0}{30}$	2.0 × 10^−5^
Depleted	Lipid Metabolism	17	$\frac{245}{713}$	1.4 × 10^−3^

Class size is the number of pathway instances for the given pathway class. The ‘Links’ column is the number of reactions among the pathways of the pathway class that have links to MetaCyc reactions, over the total number of reactions for the pathway class. The Bonferroni-corrected p-value from the hypergeometric test indicates the probability that the observed proportion of reactions with links within the pathway occurred by chance. Pathways with a p-value at or below a cut-off of *α* = 0.025 are shown. The full list may be found in the Additional file 2.

We also asked whether the pathways that are unique to MetaCyc have a taxonomic bias. MetaCyc curators associate an approximate “taxonomic range” for a pathway based on their assessment that most species within the taxonomic range are likely to contain the pathway. The Taxonomic-Range slot lists one or more particular organisms or higher-rank taxa such as phyla or kingdoms. Table 13 scores a MetaCyc pathway as unique if one-third or fewer of the pathway’s reactions have links to KEGG reactions within the same KEGG pathway. Only MetaCyc taxa with 50 or more corresponding MetaCyc pathways are included. An equivalent analysis was not performed using KEGG pathways, as they do not contain taxonomic information in their MAP or MODULE datasets.

**Table 13:** Taxonomic analysis of MetaCyc base pathways that are not represented in KEGG pathways

**ID**	**Taxon**	**Pathways**	**Unique Pathways**	**% Unique**
131567	Cellular Organisms	1840	878	47.7
2759	Eukaryota	1094	512	46.8
**33090**	**Viridiplantae (green plants)**	**650**	**348**	**53.5**
**35493**	**Streptophyta**	**374**	**222**	**59.4**
**3193**	**Embryophyta (plants)**	**373**	**221**	**59.2**
**58023**	**Tracheophyta (vascular plants)**	**298**	**181**	**60.7**
**78536**	**Euphyllophyta**	**289**	**175**	**60.6**
**58024**	**Spermatophyta (seed plants)**	**285**	**174**	**61.1**
**3398**	**Magnoliophyta (flowering plants)**	**262**	**168**	**64.1**
**91827**	**Core Eudicotyledons**	**162**	**108**	**66.7**
**71275**	**Rosids**	**100**	**71**	**71.0**
33154	Opisthokonta	351	131	37.3
33208	Metazoa (multicellular animals)	129	47	36.4
7711	Chordata	54	20	37.0
7742	Vertebrata	52	20	38.5
4751	Fungi	219	78	35.6
2	Bacteria	1040	426	41.0
**201174**	**Actinobacteria**	**72**	**37**	**51.4**
1224	Proteobacteria (purple photosynthetic bacteria)	169	64	37.9
2157	Archaea	209	82	39.2

The ID column is the NCBI Taxonomy DB [42] identifier. The pathways column is the number of MetaCyc pathways that occur in that taxon based on its Taxonomic-Range slot. The unique pathways column is the number of MetaCyc base pathways for that taxon that are unique relative to KEGG pathways. The percent unique column is the fraction of MetaCyc base pathways for that taxon that are unique relative to KEGG pathways, with rows with a fraction greater than 50% shown in bold. The rows of the table are sorted with respect to the NCBI Taxonomy. Relative taxonomic rank is indicated by indentation. Taxa of the same rank are ordered by decreasing percent unique pathways. The taxon of “Cellular Organisms” is included to provide a baseline from which to compare other taxa.

## Discussion

### Compounds

Table 1 shows that both DBs contain significant numbers of compounds that are not substrates in any reaction, e.g., 8,249 of the compounds in KEGG do not directly participate in any reaction; 3,100 MetaCyc compounds do not directly participate in any reaction. MetaCyc includes such compounds for a variety of reasons: some such compounds are activators, inhibitors, and cofactors of MetaCyc enzymes; others are analogs of reaction substrates; others are expected to be present in reactions that will be curated in the future; still others are *indirect* substrates of MetaCyc reactions because they are instances of MetaCyc compound classes that are substrates of MetaCyc generic reactions. Although users might not expect pathway DBs to contain metabolites that are not participants in pathways or reactions, these metabolites may be useful for identification of compounds from metabolomics datasets.

KEGG contains more duplicate compound entries than does MetaCyc, but overall compound duplicates are relatively low in both DBs.

MetaCyc provides a richer set of compound data fields than KEGG does, including SMILES [43] and InChI [31] strings for most compounds (SMILES is also an ASCII system for encoding chemical structures). In addition, MetaCyc compounds are cross-referenced to the enzymes for which they are activators, inhibitors, and cofactors.

KEGG contains 2.0 times more compounds with comments than does MetaCyc, but the KEGG comments are extremely short, averaging 6.5 characters per comment. MetaCyc comments average 47.7 characters in length. Many KEGG comments are single phrases such as “pesticide”.

MetaCyc contains 2.4 names per compound compared to 1.6 for KEGG, which may render MetaCyc more able to recognize chemical names in chemical datasets that use non-standard nomenclature (e.g., metabolomics datasets). On the other hand, KEGG does contain significantly more compounds than MetaCyc.

### Reactions

As noted earlier, many metabolites within the two DBs are not substrates of any reaction; similarly, many reactions within the two DBs are not components of any pathway. This situation occurs for a variety of reasons. Biologically, many metabolic reactions have not been assigned to a metabolic pathway. MetaCyc attempts to gather a comprehensive compendium of bioreactions for applications such as flux-balance analysis and design of novel metabolic pathways, that do not depend soley on reactions within defined metabolic pathways. In addition, some reactions in MetaCyc and KEGG will probably be assigned to pathways curated in the future.

Overall, MetaCyc contains 1.2 times as many reactions as does KEGG. Applications such as flux-balance analysis require reactions that are fully balanced (including hydrogen) because unbalanced reactions violate conservation of mass and thus the model can generate non-physical flux values. MetaCyc curators routinely encounter unbalanced reactions in the literature, and although many such unbalanced reactions can be corrected by curators, for some unbalanced reactions it is not clear how to correct them.

We can calculate for each DB the number of “high quality reactions” by subtracting from each total the duplicate reactions, and the unbalanced reactions. The results are MetaCyc: 9,451 and KEGG: 6,900, a ratio of 1.37:1.

MetaCyc also provides a richer set of attributes for reactions than does KEGG, such as identification of spontaneous reactions.

The atom mapping of a reaction describes for each reactant non-hydrogen atom its corresponding atom in a product compound. KEGG has provided atom-mapping data through its RPAIR attribute for several years; the Feb 2012 version of KEGG contains atom-mapping data for 8,292 reactions. MetaCyc began providing atom mapping data in version 16.5 in November 2012, which contains atom-mapping data for 8,281 reactions.

Although both DBs employ *generic reactions*, some details of the treatment of these reactions differ. Generic reactions are reactions in which one or more substrates denote a set of possible compounds, often by using R-groups. For example, the MetaCyc reaction DEOXYCYTIDINE‐KINASE‐RXN describes the reaction deoxycytidine+a nucleoside triphosphate →dCMP + a nucleoside diphosphate

KEGG contains the same reaction (R02321) with the same equation. However, KEGG represents the compound classes differently. In MetaCyc “a nucleoside triphosphate” is described by a class frame (Nucleoside‐Triphosphates). The MetaCyc ontology links that class frame to several subclasses, and ultimately to eleven specific compounds that are instances of that class, such as ATP. This representation allows software within Pathway Tools to generate instantiations of generic reactions — namely, to generate all possible instance reactions (reactions all of whose substrates are instance compounds, not classes) that are specializations of the generic reaction. MetaCyc contains 2,884 generic reactions, from which many additional reactions can be generated through instantiation. In contrast, although KEGG contains an object representing the generic compound (C00201), that generic compound is not found in the KEGG BRITE ontology, nor does KEGG contain links from the generic compound to instances of that compound. Thus, so far as we know, the KEGG representations do not facilitate programmatic instantiation of generic reactions.

### Pathways

Based on Table 7, MetaCyc contains 10.3 times as many base pathways as KEGG contains modules. MetaCyc contains 1.2 times as many superpathways as KEGG contains maps. Because pathway size measured in reactions varies so strongly between the two DBs, comparing the DBs purely based on pathway counts can be misleading — the average MetaCyc base pathway contains 4.37 reactions, whereas the average KEGG map contains 28.84 reactions. Furthermore, 17% of MetaCyc pathways consist of a single reaction step — namely, in those cases where the MetaCyc curation rules on defining pathway boundaries [44,45] result in single-reaction pathways.

A more meaningful way to compare the pathway complements of the two DBs is to compare the size of the metabolite and reaction spaces covered by these pathways. Table 1 shows that MetaCyc pathways refer to 5,523 distinct metabolites, or 1.16 times as many as KEGG. A small difference exists between the substrates covered by MetaCyc base pathways versus MetaCyc super pathways, most likely because MetaCyc super pathways are ultimately defined in terms of base pathways, plus some additional reactions not present in the base pathways. In contrast, there is a large difference between the substrates covered by KEGG maps versus modules — modules cover a very small set of substrates compared to maps and compared to MetaCyc pathways.

We posit that KEGG has such a small number of modules because modules were introduced to KEGG in the last few years, and their coverage is still limited. For example, KEGG contains one module for proline biosynthesis; MetaCyc contains four such pathways. KEGG lacks modules for biosynthesis of the amino acids valine, glycine, aspartate, alanine, glutamine, and glutamate (most but not all are one reaction pathways). That MetaCyc contains 10.3 times as many base pathways as KEGG contains modules means that studies such as [46] that analyze the pathway content of metagenomic samples may be incomplete because they may miss pathways using the limited repertoire of KEGG modules that could be found using MetaCyc base pathways (note that [46] also included KEGG maps in their analysis).

MetaCyc pathways refer to 6,348 reactions, or 1.03 times as many reactions as referred to in KEGG pathways. Thus, the reaction spaces covered by the two DBs are very similar in size.

MetaCyc provides a more extensive array of pathway attributes than does KEGG. Some of these attributes can be used to increase the accuracy of pathway prediction, e.g., Taxonomic-Range and Key-Reactions. Lacking those attributes, pathway predictions performed using KEGG pathways are likely to be less accurate than for MetaCyc pathways.

In the years before KEGG introduced its modules, KEGG and MetaCyc employed very different conceptualizations of pathways. As discussed in detail in [45], KEGG maps are larger than MetaCyc base pathways because KEGG maps are mosaics that integrate reactions from multiple organisms and multiple biological pathways. For example, KEGG map00270 (“cysteine and methionine metabolism”) integrates reactions from pathways involving the biosynthesis of both L-cysteine and L-methionine, and their conversion to compounds such as L-cystathionine and L-homocysteine, from all domains of life. In contrast, MetaCyc creates separate base pathways — called pathway *variants* — for each distinct pathway of L-methionine biosynthesis (eight pathways) and L-cysteine biosynthesis (four pathways) that has been experimentally elucidated in a given organism (pathways are considered distinct if they contain different sets of reactions). MetaCyc pathway boundaries are defined [45] based on evolutionary conservation, on the metabolism literature, on regulation, and on stable high-connectivity metabolites. We estimate that KEGG modules are created according to principles similar to those of MetaCyc base pathways.

These differences in pathway conceptualization have different implications, depending on the intended uses of pathway data. (1) MetaCyc pathways (and probably KEGG modules) more accurately portray the exact biological pathways that occur in a specific organism, because for a KEGG map, its mosaic nature means that the user cannot tell which subset of its reactions was experimentally elucidated in a particular organism. (2) KEGG maps (and MetaCyc superpathways) are more effective at portraying the set of possible reactions that can impinge on a given metabolite in a wide range of organisms. (3) KEGG maps are not effective for statistical correlation studies because they encompass so much metabolic ground. For example, if we compare two metagenomic datasets and find that map00270 (“cysteine and methionine metabolism”) is present in one but not the other, is it the biosynthesis of cysteine that is over represented, or that of methionine? Or is it the biosynthesis of other compounds in this map (such as L-cystathionine and L-homocysteine) that are over represented? Abubucker et al. make a similar point [46] about KEGG maps. (4) We argue that for pathway reconstruction in sequenced genomes, MetaCyc pathways are more effective because their smaller size produces more focused predictions. For example, KEGG shows its map00680 (“methane metabolism”) as present in *E. coli* K-12 MG1655 with 23 reactions (excluding transporters) colored as occurring in this organism. Yet, *E. coli* K-12 MG1655 does not produce methane. A counter-example of KEGG pathway prediction comes from the photosynthesis map (map00195), for which only annotations based on photosynthetic organisms can be selected on the KEGG website. Thus, it is unclear what rules KEGG uses to call a given map as present or absent in a given organism; the rules used by Pathway Tools are published [47].

When a map is called as present by KEGG, does it predict all reactions in the map as present in that organism? For example, for the methane metabolism pathway in *E. coli*, are the additional 55 uncolored reactions inferred as present in textitE. coli? Since KEGG pathways are known to be multi-organism mosaics, such an inference will surely contain many false-positive reactions. In contrast, when a MetaCyc pathway is predicted as present, the assertion is that all of its reactions are probably present, permitting a more focused and accurate prediction of the reactome of an organism. This more accurate prediction of the reactome has implications for metabolic modeling using flux-balance analysis, where missing reactions usually yield non-solvable models, whereas extra reactions can yield models that make erroneous predictions. KEGG may resolve these issues once its collection of modules is more extensive, but currently its modules cover too little of metabolism to have broad utility.

Figure 1 and Figure 2 reveal that while MetaCyc base pathways have a distribution range comparable to that of KEGG modules, there is a significant difference in mean and variance for MetaCyc super pathways and KEGG maps. Many pathway analyses, such as enrichment/depletion, may exhibit bias when the sets of pathways have a large range of sizes. By virtue of having a smaller range of sizes, MetaCyc super pathways provide a more consistent basis for performing pathway analyses.

We analyzed the degree of overlap on a pathway-class basis in Tables 11 and 12, revealing the pathway classes that are enriched for reaction links (i.e., there is a significant amount of overlap between the two databases), and the pathway classes that are depleted for reaction links (i.e., the pathway class is relatively unique to its database). The KEGG pathway class depletion in Table 12 shows that the metabolism of MetaCyc is under-represented for counterparts of the KEGG maps for xenobiotics, glycans, and polyketides. For glycans and polyketides, we expect that this is because MetaCyc does not currently have the ability to represent abstracted versions of glycan chemical structures, nor abstracted versions of polyketide pathways, found in KEGG map drawings.

Table 13 shows that MetaCyc contains large numbers of unique pathways, which are primarily found in plant taxa, but are also found in vertebrates, chordata, and metazoa; in fungi; in archaea; and in proteobacteria.

### Miscellaneous

MetaCyc contains extensive data on metabolic enzymes. Version 16.0 of MetaCyc contains 7,893 metabolic enzymes. MetaCyc describes enzyme subunit composition, substrate specificity, activators, inhibitors, and cofactor requirements. KEGG does not describe the protein properties of metabolic enzymes, and therefore lacks this type of data; KEGG does associate cofactors with reactions.

MetaCyc and KEGG also differ in their licensing terms. MetaCyc data are freely available to all users *via* data file download in multiple formats, and may be openly redistributed. KEGG dataset FTP downloads are available for a fee to all users, and may not be openly redistributed. KEGG provides a web service API for requesting entries individually, as does MetaCyc.

## Conclusions

We have compared the contents of the KEGG and MetaCyc pathway DBs. Because pathway DBs contain multiple types of data, our comparison is necessarily multidimensional. KEGG contains significantly more compounds than does MetaCyc, whereas MetaCyc contains significantly more reactions and pathways than does KEGG. However, the number of reactions occurring in pathways, 6,348 for MetaCyc and 6,174 for KEGG, are quite similar. Only 1,961 of those reactions have been identified as reactions shared by the two DBs. We expect that as many as 1,636 additional shared reactions will be found, which would leave a substantial number of reactions that are unique to each DB. We estimated the set of pathways that are present in one DB but not in the other DB, and found that MetaCyc pathways not found in KEGG are predominately from plants, fungi, metazoa, and actinobacteria; KEGG pathways not found in MetaCyc are for xenobiotic degradation, glycan metabolism, and metabolism of terpenoids and polyketides. MetaCyc contains more reactions that are fully balanced, which facilitates metabolic modeling such as using flux-balance analysis. MetaCyc includes generic reactions that may be instantiated computationally. Both databases contain atom-mapping data.

## Competing interests

The authors benefit financially from commercial licensing of the Pathway Tools software.

## Authors’ contributions

TA authored substantial text, and performed most of the comparisons and analyses. MT authored the KeggCyc loader software. AK curated and checked many correspondences between KEGG and MetaCyc. RC commented on the manuscript and provided valuable discussions on many issues. PDK supervised the research and authored substantial text. All authors read and approved the final manuscript.

## Supplementary Material

Additional file 1 The software used in building the KeggCyc DB is available for use with Pathway Tools as a Common Lisp source code file. Click here for file

Additional file 2 The full results of the enrichment/depletion analysis may be found in the additional file in a spreadsheet file. Click here for file
